# Synergistic Integration of Superhydrophobic and Lubricant-Infused Surface for Enhanced Liquid Repellency

**DOI:** 10.1021/acs.langmuir.5c02261

**Published:** 2025-08-27

**Authors:** Hong-Ren Jiang, I-Chen Wen, Kuei-Wen Wang, Pin-Xu Ko, Bo-Wei Li

**Affiliations:** Institute of Applied Mechanics, 33561 National Taiwan University, No.1, Sec. 4, Roosevelt Rd., Da’an Dist., Taipei 106, Taiwan (R.O.C.)

## Abstract

We present a novel dual-functional surface design that strategically integrates superhydrophobic and lubricant-infused surface technologies to achieve switchable liquid repellency with significantly enhanced durability and self-healing capabilities. By precisely controlling the amount of silicone oil infused—quantified as surface loading—into laser-induced graphene structures on polyimide substrates, we demonstrate a controlled transition between superhydrophobic and lubricant-infused states. This hybrid approach effectively addresses the critical challenges of both technologies: the mechanical vulnerability of superhydrophobic surfaces and lubricant depletion issues in lubricant-infused surfaces. Our systematic investigation reveals an optimal silicone oil loading that maintains excellent water repellency even after substantial surface damage, evidenced by high contact angles and low sliding angles across damaged regions of up to 2 mm in width. The self-healing mechanism provided by the strategic lubricant layer preserves liquid-repellent properties after physical damage, significantly outperforming conventional superhydrophobic surfaces in durability tests. Experimental results using candle-soot-treated surfaces further confirm the superior damage resistance of our hybrid design. This work provides new insights into liquid-repellent surface engineering with potential applications in self-cleaning coatings, anti-icing surfaces, oil–water separation, and microfluidic devices that require sustained performance in harsh environments.

## Graphical abstract

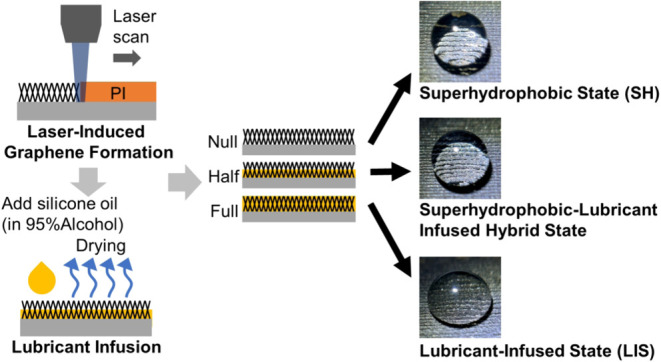

## Introduction

Liquid-repellent surfaces^1^ have attracted significant attention due to their broad applications in self-cleaning materials, anti-icing surfaces, oil–water separation, and microfluidic devices.^2–4^ Among various strategies, superhydrophobic (SH) surfaces and lubricant-infused surfaces (LIS) represent two major approaches to achieving liquid repellency.^2–5^ However, both technologies face distinct challenges that limit their practical applications.

Superhydrophobic surfaces, inspired by natural phenomena such as the lotus leaf effect,^6^ typically rely on micro- and nanoscale surface structures combined with low surface energy materials.^7^ While contact angles exceeding 150° are commonly cited, superhydrophobic surfaces are fundamentally characterized by the Cassie–Baxter wetting state with stable air entrapment.^6,8–11^ These surfaces exhibit excellent water repellency; however, they suffer from poor mechanical durability and limited self-healing capabilities.^1,7^ Furthermore, their effectiveness significantly deteriorates under harsh conditions, particularly when exposed to low surface tension liquids or high humidity environments.^2,12,13^

Lubricant-infused surfaces, introduced as an alternative approach, demonstrate superior stability and broader liquid repellency.^3,5^ These surfaces maintain their functionality through a stable lubricant layer trapped within surface structures, enabling effective repellency against various liquids and self-healing properties.^3,14^ Their versatile performance has led to promising applications in waterproofing, anti-icing surfaces, oil–water separation, and drag reduction systems.^15–18^ However, the gradual depletion of lubricant through evaporation, shear forces, or gravity poses a significant challenge for long-term applications.^3,14,19^

Recent advances in liquid-repellent surface research have focused on addressing these fundamental limitations through innovative design strategies and enhanced fabrication approaches. Contemporary studies have increasingly emphasized the critical importance of mechanical robustness and long-term environmental stability for practical applications.^20,21^ Furthermore, recent investigations into bioinspired design principles have provided new insights into optimizing surface topographies for improved lubricant retention and reduced depletion rates.^22^ These efforts have focused on either enhancing the mechanical durability of superhydrophobic surfaces^1,12,23^ or improving lubricant retention in LIS.^3,22^ While previous work has shown that superhydrophobic surfaces can be transformed into lubricant-infused surfaces through controlled oil infiltration,^24^ such approaches primarily focused on complete state conversion rather than investigating the synergistic benefits of maintaining both repellency mechanisms simultaneously. However, few studies have explored the potential synergistic effects of integrating these two approaches.^25–28^ The integration of superhydrophobic and lubricant-infused technologies could potentially combine their respective advantages, while mitigating their individual limitations. In this work, we present a novel surface design that synergistically integrates superhydrophobic and lubricant-infused surface technologies. Our approach enables tunable liquid repellency through precise control of lubricant infusion into hierarchically structured surfaces. The design features a unique architecture in which a lubricant layer is strategically infused beneath a trapped air layer, ensuring sustained repellency even when the superhydrophobic state is compromised. This dual-functional surface not only maintains excellent liquid repellency under harsh conditions but also demonstrates enhanced durability and self-healing capabilities.

We systematically investigate how different levels of lubricant loading influence the wetting properties, identifying an optimal range that maintains the superhydrophobic character while self-healing capabilities. Through controlled surface damage experiments, we demonstrate that the integrated design significantly outperforms conventional superhydrophobic or singular lubricant-infused surfaces in terms of durability and sustained repellency. The synergistic interaction between the trapped air layer and the infused lubricant provides a redundant repellency mechanism, where damage to the superhydrophobic state triggers a localized transition to a lubricant-infused state that preserves the liquid repellency.

## Experimental Methods

### Materials

Polyimide Tape (Kapton, DuPont, 125 μm thickness) was used as the substrate for the laser-induced graphene formation. Silicone oil (viscosity: 10 cSt, Sigma-Aldrich) was selected as the lubricating fluid due to its low surface tension, excellent chemical stability, and appropriate viscosity. Ethanol (95%) was used to prepare the silicone oil mixture for the controlled infiltration. For the control experiments, candle soot was collected from commercial paraffin candles. Deionized water (resistivity >18.2 MΩ·cm) was used for all wetting measurements.

### PDMS and TiO_2_–PDMS Silicone Oil Infusion Process

PDMS prepolymer and curing agent were mixed at a weight ratio of 10:1, followed by degassing in a vacuum chamber for 30 min to remove air bubbles. The mixture was then poured into a mold and cured in an oven at 70 °C for 4 h to form PDMS films approximately 2 mm thick. Cured PDMS (Sylgard 184, Dow Corning) samples were immersed in silicone oils at room temperature for 24 h to ensure complete infiltration. After removal, the samples were gently wiped with lint-free tissue to remove excess surface oil, resulting in silicone oil-infused PDMS.

The TiO_2_–PDMS system was prepared using TiO_2_ nanoparticles (Advanced Ceramics Nanotech, rutile, 40 nm) with minimal PDMS binder. A suspension containing 20 wt % TiO_2_ and 1 wt % gum arabic was applied onto a glass slide in three sequential coats using a 150 μm spacer, with drying between each layer to ensure uniform deposition. Subsequently, the PDMS prepolymer and curing agent were diluted in *n*-hexane at a weight ratio of 2:1:200, and 300 μL of the mixture was applied onto the coated surface, followed by heat-curing to create a porous ceramic structure. The surface was then treated with silicone oil and heated at 200 °C for hydrophobicity surface modification,^29^ followed by final silicone oil infusion and removal of excess oil using the gravity tilting method.

### Laser-Induced Graphene Surface Fabrication

Hierarchical superhydrophobic surface structures were fabricated by using a programmable laser engraving system (FLUX Lazervida, 10W, 445 nm). The laser parameters were optimized with a power output of 0.83 W, a scanning speed of 17 mm/s, and a focused beam diameter of approximately 100 μm. The laser scanned the entire surface area (1.5 × 1.5 cm^2^) with a line spacing of 100 μm to ensure uniform coverage. During laser irradiation, the localized high temperature induced the photothermal transformation of polyimide into porous graphene structures.

### Lubricant Infusion into the LIG Surface

Silicone oil (viscosity: 10 cSt) was selected as the lubricating fluid. A 95% ethanol and silicone oil mixture (19:1 weight ratio) was prepared, with 40 μL applied to the surface per treatment. Samples were oven-dried (70 °C) for 5 min to accelerate ethanol evaporation, and this process was repeated until the total oil loading on the surface reached the desired level (0–17.3 mg/cm^2^). The samples were then left to rest for 1 day to allow uniform distribution of the oil. To validate actual oil retention and remove any loosely bound excess lubricant, all oil-treated samples were subsequently tilted at 90° for 12 h. Gravimetric analysis using a precision analytical balance (±1 mg accuracy) revealed minimal weight loss within the measurement error range after the gravity-induced shedding process.

### Preparation of Candle-Soot-Based SH Surfaces

For comparative studies, candle-soot-based superhydrophobic surfaces were prepared by holding a clean glass slide over a paraffin candle flame until a uniform black soot layer was deposited. For lubricant-infused samples, after the first soot layer deposition, 30 μL of a 95% ethanol and silicone oil mixture (500:1 weight ratio) was applied to the surface and allowed to dry completely for ethanol evaporation, followed by a second soot layer deposition. For the SH surface, two soot deposition steps were similarly performed but without silicone oil addition. The total candle soot deposition time was kept identical for both sample types to ensure a comparable soot layer thickness and structure.

### Surface Morphology Analysis

Surface morphology was characterized by using a scanning electron microscope (Hitachi TM-3000). Samples were directly measured without an oil infusion.

### Wetting Characterization

Contact angle measurements were performed by using a camera at room temperature (25 ± 2 °C). A water droplet (20 μL) were deposited on the surface for each measurement. Sliding angles were measured by tilting the substrate at 1°/s. At least three measurements were taken at different locations per sample, with a minimum of three samples analyzed to ensure reproducibility.

### Durability and Self-Healing Performance Evaluation

Two damage tests were conducted to evaluate the surface durability.

For compression damage, we applied a finger pressure of approximately 10N to the LIG surface. Due to the nonelastic nature of LIG, this localized compression resulted in compression-induced fissures on the surface.

For controlled strip damage, we utilized a fiber laser engraving system operating at a 1064 nm wavelength. The laser parameters were optimized with a power output of 6 W, a scanning speed of 1000 mm/s, and a focused beam diameter of approximately 50 μm. This higher-power laser effectively removed the graphene layer within the targeted regions. We applied one to two passes until the graphene layer was visibly removed from the scanned areas. Strip damages of varying widths (0.1, 1, and 2 mm) were created on different samples to systematically investigate the relationship between damage scale and surface functionality.

### Durability Evaluation

The durability of the surfaces was evaluated through continuous water droplet impact tests. A custom-built setup was used where water droplets (45 μL) were released from a fixed height of 1.2 cm onto the same location of the sample surface at a rate of 1.2 droplets per second.

## Results and Discussion

We successfully fabricated dual-functional liquid-repellent surfaces by integrating laser-induced graphene (LIG)^30–32^ structures with controlled silicone oil loading. The fabrication process, schematically illustrated in Figure 1a, involves laser ablation of a polyimide (PI) substrate to generate hierarchical LIG structures,^33–35^ followed by precise infusion of silicone oil. Scanning electron microscopy (SEM) analysis of the LIG surface prior to oil infusion revealed a characteristic porous and hierarchical morphology (Figure 1b). This structure, formed by the photothermal conversion of PI, consists of interconnected micro- and nanoscale graphene flakes, creating ample void spaces. This inherent porosity is crucial for both trapping air to establish superhydrophobicity (Cassie–Baxter state) and retaining the infused lubricant for the self-healing functionality.

**Figure 1:**
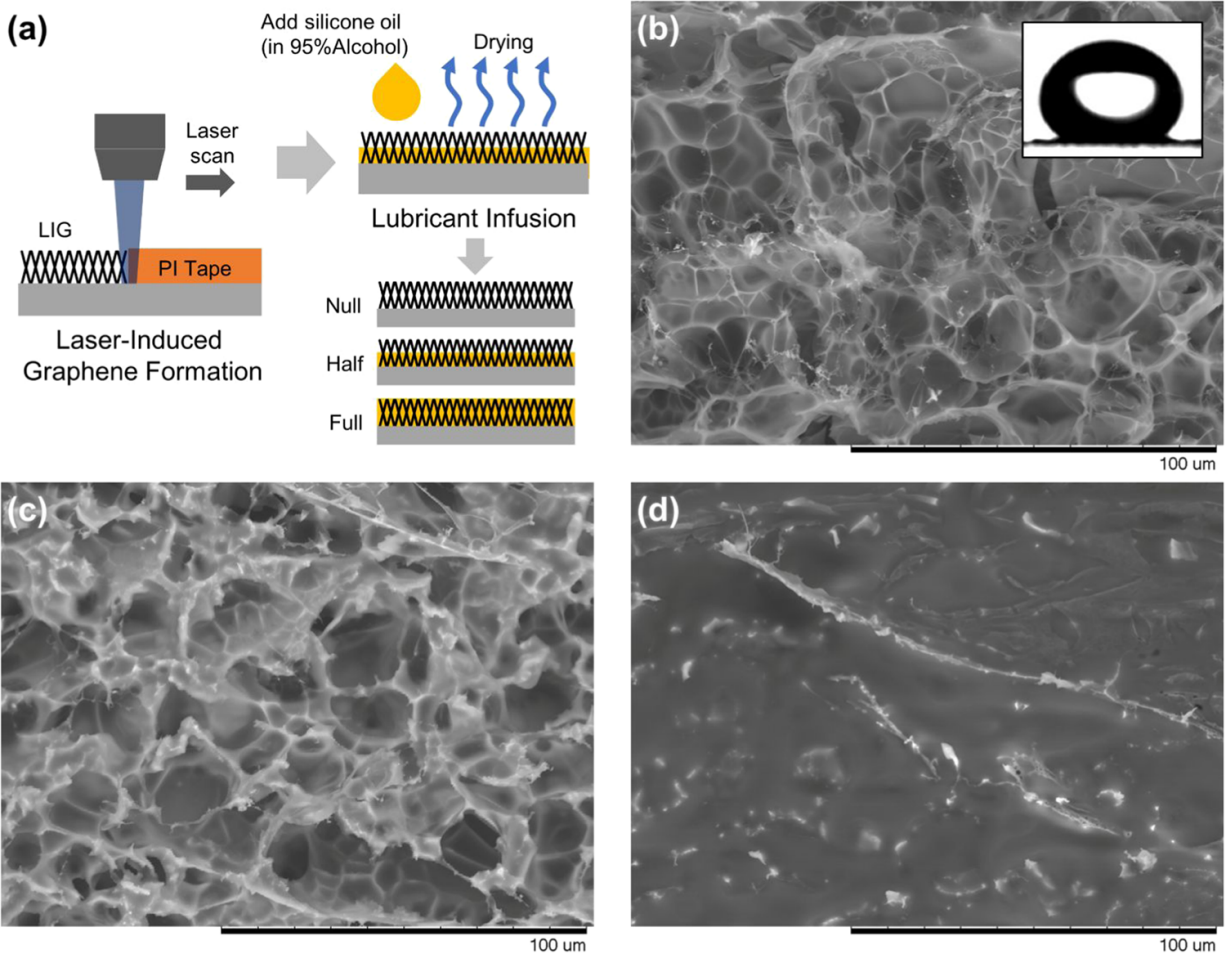
Fabrication and characterization of laser-induced graphene (LIG) superhydrophobic surfaces with controlled silicone oil infusion. (a) Schematic illustration of the fabrication process, showing blue laser scanning (445 nm) of polyimide film followed by silicone oil infusion and the effect of varying silicone oil loading on the resulting surface properties. (b) The SEM micrograph of the LIG surface showing the characteristic protrusions created by laser ablation; the inset shows the superhydrophobic behavior with a water droplet exhibiting a high contact angle. (c) The SEM image of the LIG surface with moderate silicone oil loading (2.9 mg/cm^2^) demonstrating oil deposition while maintaining unfilled void spaces that preserve air pockets essential for hybrid functionality. (d) The SEM image of the LIG surface with high silicone oil loading (10.8 mg/cm^2^) showing substantial filling of the micro- and nanoscale topography.

To visualize the oil distribution after infusion, we employed curable diluted PDMS as a model lubricant, which shares the same chemical composition as the silicone oil used in our experiments. At moderate oil loading (2.9 mg/cm^2^), SEM imaging revealed that a lubricant layer was deposited on the porous surface while maintaining unfilled void spaces that preserve air pockets essential for the Cassie–Baxter state (Figure 1c). However, at higher oil loading (10.8 mg/cm^2^), the surface topography became substantially filled, with most micro/nanoscale features obscured by the excess lubricant layer (Figure 1d). The effect of varying silicone oil loading on the overall surface properties demonstrates a transition between different wetting regimes, ranging from a predominantly air-pocket-dominated surface at low oil loading to a lubricant-dominated surface at high oil loading.

Silicone oil loading significantly influences the surface wetting behavior, enabling a transition between superhydrophobic and lubricant-infused states. We systematically investigated this transition by measuring the static water contact angle (CA) and sliding angle (SA) as a function of silicone oil loading (mg/cm^2^), as shown in Figure 2a, with the corresponding optical images of CA and SA measurements presented in Figure 2b. The pristine LIG surface (0 mg/cm^2^ oil) exhibited excellent superhydrophobicity with a high CA of approximately 143° and a low SA below 6.8°, indicating a stable Cassie–Baxter state, where water droplets reside on trapped air pockets within the LIG structure.

**Figure 2:**
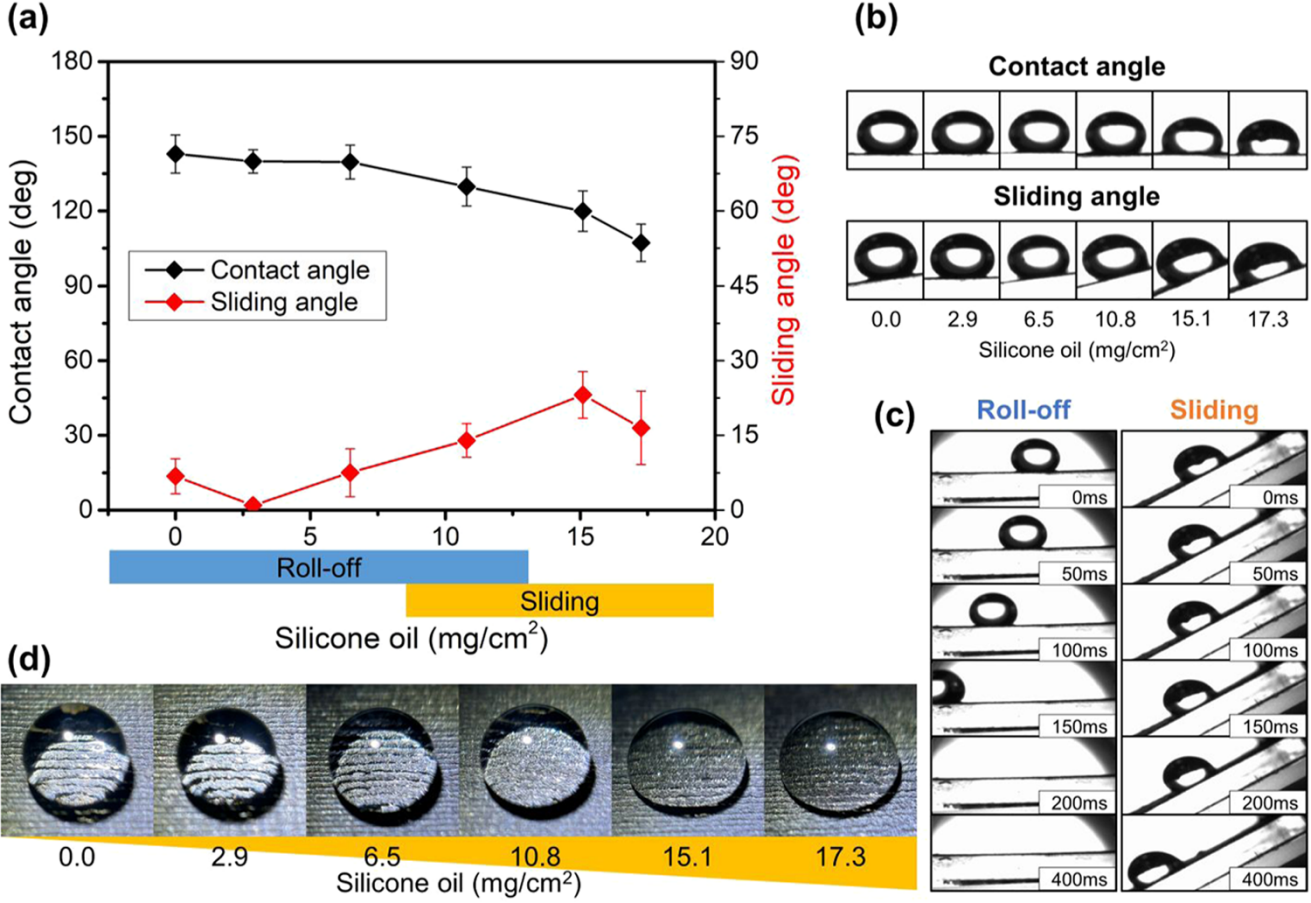
Influence of silicone oil loading on surface wetting properties. (a) Quantitative relationship between silicone oil loading (mg/cm^2^) and the resulting water contact angles (°) and sliding angles (°), showing the transition from superhydrophobic to lubricant-infused behavior with increasing oil loading. (b) Optical images showing contact angle and sliding angle measurements across all tested silicone oil loading conditions. (c) Visual comparison demonstrating two distinct droplet mobility mechanisms: rolling behavior on low oil loading (2.9 mg/cm^2^) surfaces versus sliding behavior on high oil loading (17.3 mg/cm^2^) surfaces. (d) Optical images of water droplets on LIG surfaces demonstrating how varying silicone oil loading influences air layer retention beneath the droplets. Greater air retention is observed at lower oil loading, maintaining the Cassie–Baxter state with enhanced robustness.

Upon infusion with a low silicone oil loading (e.g., 2.9 mg/cm^2^), the surface largely retained its high CA (∼140°) and low SA (∼0.9°). This suggests that at low oil loading, the oil primarily occupies the base of the LIG pores or forms a very thin layer, preserving significant air pockets that maintain the Cassie–Baxter state and characteristic low-adhesion, “roll-off” behavior for water droplets (Figure 2c, left panel). As the oil loading increased further (e.g., 6.5 mg/cm^2^), the CA remained high (∼140°), while the SA showed a noticeable increase (∼7.5°), transitioning toward typical values observed for LIS. At higher oil loading (e.g., 10.8 mg/cm^2^ and above), the SA plateaued around 20–30°, and the droplet mobility mechanism shifted from rolling to sliding (Figure 2c, right panel). This transition indicates that the LIG structure becomes progressively filled with lubricant, replacing the air pockets. While still highly repellent, the surface dynamics change as the water droplet now interacts primarily with the smooth liquid lubricant layer rather than with a composite air–solid interface. Figure 2d illustrates how increasing the oil loading gradually diminishes the trapped air fraction, eventually leading to a fully lubricant-infused state. Optical images of contact angles and sliding angles under each silicone oil loading condition provide visual confirmation of this wetting behavior transition. An optimal balance appears around 2.9 mg/cm^2^, where high static repellency (high CA) and good dynamic repellency (low SA) coexist, suggesting the presence of both air pockets and a stabilizing-healing lubricant layer.

A key objective of this hybrid design is to overcome the mechanical fragility of conventional superhydrophobic (SH) surfaces. We first tested the surface robustness by applying compressive pressure to induce damage on surfaces with varying silicone oil loading. This compression primarily resulted in surface fissures. As shown in Figure 3a,b, contact angle measurements revealed no significant changes regardless of damage state, but sliding angle behavior exhibited marked differences. Surfaces without silicone oil showed substantially increased sliding angles after damage, with water droplets becoming pinned to the surface. In contrast, surfaces with silicone oil maintained low sliding angles, effectively preventing droplet pinning, even after damage.

**Figure 3:**
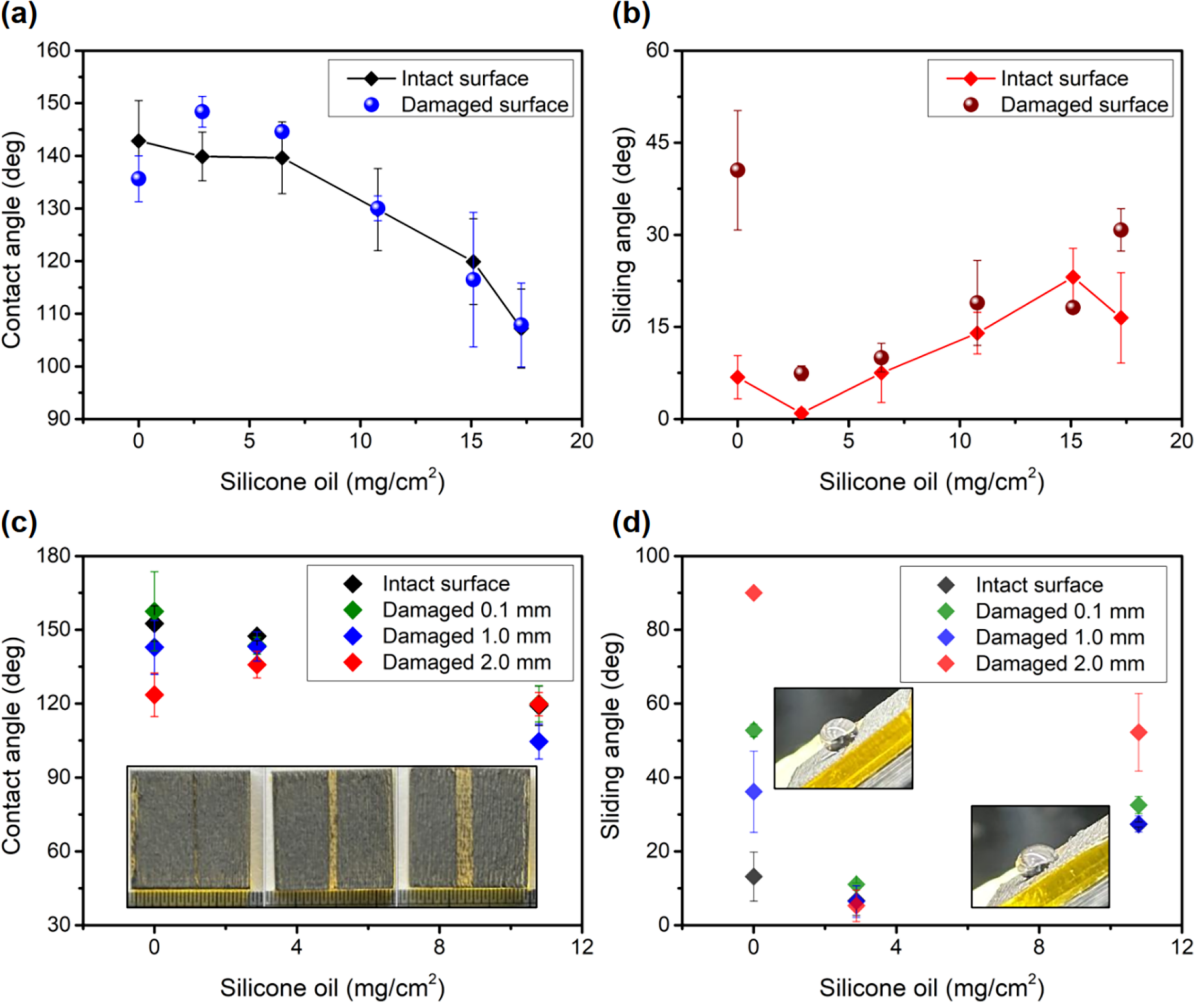
Mechanical robustness evaluation of hybrid surfaces with varying silicone oil loading. (a) Water contact angle measurements before and after compression damage on surfaces with different silicone oil loading. (b) Sliding angle measurements comparing intact and compression-damaged surfaces. (c) Water contact angles measured on surfaces with controlled laser-ablated strip damage of varying widths (0.1, 1, and 2 mm); the inset shows optical images of the damaged LIG surfaces. (d) Sliding angle measurements on surfaces with laser-ablated strip damage; the upper inset shows water droplet pinning on the damaged region without silicone oil; the lower inset shows droplet behavior with excessive oil loading (10.8 mg/cm^2^); optimal performance is observed at moderate oil loading (2.9 mg/cm^2^) (see Supporting Video S1 for dynamic visualization).

We further evaluated damage resistance by introducing controlled laser-ablated stripes of varying widths onto surfaces with different oil loadings and measuring the resulting wetting properties. As shown in Figure 3c, the static contact angle remained high (>120°) across all tested oil loading and damage widths up to 2 mm. This indicates that even significant localized damage does not lead to catastrophic wetting or loss of overall hydrophobicity. However, the sliding angle measurements revealed crucial differences in dynamic repellency and highlighted the benefit of optimal lubricant infusion (Figure 3d and Supporting Video S1). On the damaged LIG surface without oil (0 mg/cm^2^, Figure 3d, higher inset), water droplets strongly pinned at the damaged site, resulting in very high sliding angles, consistent with an irreversible transition to a high-adhesion Wenzel state upon destruction of the Cassie–Baxter structure. Similarly, surfaces with very high oil loading (e.g., 10.8 mg/cm^2^) also showed increased pinning or significantly higher sliding angles on damaged regions (Figure 3d, lower inset), although they still performed better than oil-free surfaces.

Remarkably, the surface infused with the optimal oil loading (2.9 mg/cm^2^) demonstrated exceptional performance. Even when the droplet was placed on a 2 mm wide damaged stripe, the SA remained relatively low (lower than 10°). This value is significantly lower than that of the oil-free damaged surface and indicates preserved droplet mobility. This superior damage tolerance strongly suggests a self-healing mechanism mediated by the lubricant. Here, “self-healing” refers to the surface’s ability to automatically restore liquid repellency after physical damage. Upon disruption of the LIG structure within the ablated stripe, the strategically stored silicone oil from the surrounding intact porous network spontaneously wicks or flows into the compromised region. This lubricant migration effectively creates a localized LIS state precisely where the superhydrophobic structure has failed. Consequently, instead of irreversibly pinning to the higher-energy-damaged substrate (characteristic of the Wenzel state transition on the oil-free surface), the water droplet encounters this smooth, low-energy liquid interface, maintaining its mobility and preventing catastrophic wetting. This localized transition preserves the overall liquid repellency of the sample. The optimal oil loading (2.9 mg/cm^2^) provides sufficient lubricant for this localized healing without overly compromising the initial SH properties or leading to excessive lubricant coverage that might increase initial adhesion.

To further validate the enhanced durability conferred by lubricant infusion, we conducted continuous water droplet impact tests using candle-soot-based SH surfaces as a model system (Figure 4a). While LIG surfaces are inherently more robust than soot, this experiment serves to illustrate the general principle of lubricant-mediated durability enhancement for fragile soot SH structures. As shown in Figure 4b (left panels) and Supporting Video S2, the conventional candle soot SH surface (without oil) failed rapidly under repeated droplet impacts. Significant pinning was observed after ∼20 drops, and catastrophic failure with visible soot layer detachment occurred after 30 drops, rendering the surface hydrophilic. In stark contrast, the lubricant-infused candle soot surface maintained water repellency after 30 impacts (Figure 4b, right panels and Supporting Video S2). Although some minor disruption of the soot layer was visible at the direct impact location of the droplet, the droplet continued to roll or slide off easily, demonstrating the protective and potentially replenishing effect of the infused lubricant layer against mechanical stress induced by droplet impact. This result further supports the hypothesis that integrating a lubricant reservoir enhances the operational lifetime of repellent surfaces, particularly those based on delicate micro- and nanostructures.

**Figure 4:**
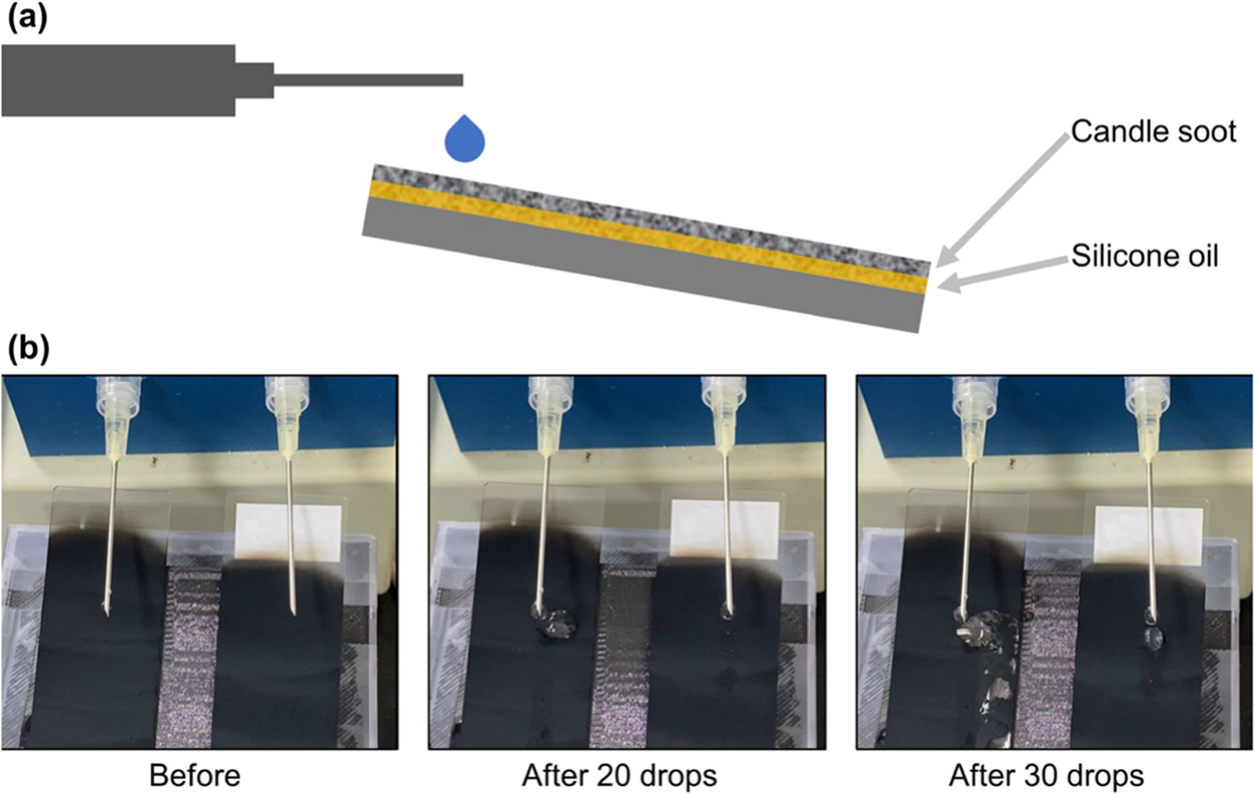
Enhanced durability of silicone oil-infused candle soot superhydrophobic surfaces under continuous water droplet impact. (a) Experimental setup showing the water droplet impact test configuration. (b) Comparative time-lapse images demonstrating the progressive degradation of surfaces under repeated water droplet impact: right panels show the oil-infused surface maintaining water repellency despite partial removal of the soot layer; left panels show the rapid deterioration of the noninfused superhydrophobic surface, with visible water droplet adhesion after approximately 20 impacts and catastrophic failure with significant soot layer detachment after 30 impacts (see Supporting Video S2 for dynamic visualization).

To confirm the effectiveness of integrating superhydrophobic and lubricant-infused functionalities into LIG surfaces, we performed high-speed water jet impact tests using 1.6 m/s water jets on surfaces tilted at 20° (Figure 5a). For pristine LIG superhydrophobic surfaces, water jet impact caused partial loss of the underlying air layer, with large water droplets remaining after impact (Figure 5b and Supporting Video S3). In contrast, the LIG surface with silicone oil (6.5 mg/cm^2^) showed no air layer loss and no droplet residue (Figure 5c and Supporting Video S4). We also compared LIG surfaces with silicone oil with PDMS-based^36,37^ and TiO_2_-based lubricant-infused surfaces. The PDMS-oil surface and TiO_2_-based lubricant-infused surface showed decreased sliding velocity and pinning after approximately 30 and 60 impacts, respectively (Supporting Video S5, Supporting Video S6), while the LIG-oil surface maintained excellent mobility after 2500 impacts without pinning (Supporting Video S7, Supporting Video S8). This demonstrates that the hybrid approach avoids rapid lubricant depletion common in conventional LIS surfaces due to intensive liquid–liquid contact. We also conducted qualitative anti-icing experiments (Figure 5d) where surfaces were cooled to −10 °C under 60% relative humidity conditions for 1 h to evaluate ice crystal formation patterns. Detailed examination of ice crystal morphology revealed distinct formation patterns between the two surface types. On oil-infused LIG surfaces (Figure 5e), ice crystals formed as discrete, three-dimensional structures with minimal surface contact, facilitated by the intervening lubricant layer that prevented direct ice-substrate adhesion. In contrast, pristine LIG superhydrophobic surfaces (Figure 5f) promoted the formation of relatively flat, surface-conforming ice layers that anchored to the micro- and nanoscale features. This morphological difference correlates with the ease of ice removal observed in our experiments, where discrete ice crystals on oil-infused surfaces could be removed more easily with minimal residue, while ice formations on pristine LIG surfaces were more difficult to remove and left residual ice deposits.

**Figure 5:**
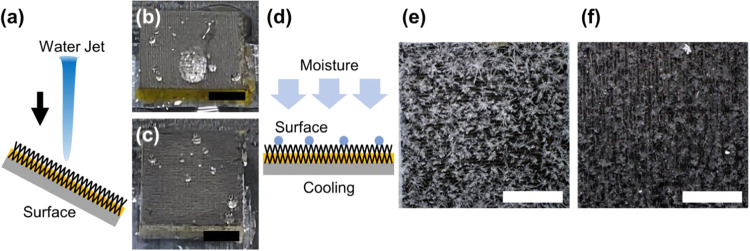
Performance evaluation of LIG hybrid surfaces under harsh conditions. (a) Schematic illustration of a high-speed water jet impact test setup with a 1.6 m/s water jet impacting the tilted surface. (b) The pristine LIG superhydrophobic surface after water jet impact, showing large water droplet residue and partial loss of the air layer. (c) The LIG surface with silicone oil after water jet impact demonstrating no droplet residue and maintained surface integrity. (d) Schematic illustration of the anti-icing experimental setup showing moisture condensation and the cooling process on the surface. (e) Ice formation on an oil-infused LIG surface showing discrete, three-dimensional ice crystals that are easily removable. (f) Ice formation on the pristine LIG surface showing flat, surface-conforming ice crystals with stronger adhesion. Scale bars: 5 mm.

Based on these findings, we propose a synergistic mechanism for our dual-functional hybrid surface, as illustrated in Figure 6. Conventional SH surfaces (Figure 6a, left) rely solely on a trapped air layer (Cassie–Baxter state). Physical damage destroys this structure, leading to an irreversible transition to the Wenzel state with high adhesion and loss of repellency (Figure 6a, right). Our optimally lubricant-infused hybrid surface (Figure 6b, left) initially exists primarily in a Cassie–Baxter state, possibly stabilized by a thin lubricant film, exhibiting high CA and low SA. Upon damage, the LIG structure is locally compromised. However, instead of transitioning to a Wenzel state, the stored silicone oil from the surrounding intact porous structure rapidly migrates to the damaged area. This creates a localized LIS state (Figure 6b, right), effectively “healing” the damage by presenting a low-energy liquid interface to the water droplet, thus maintaining low sliding angles and preserving repellency.

**Figure 6:**
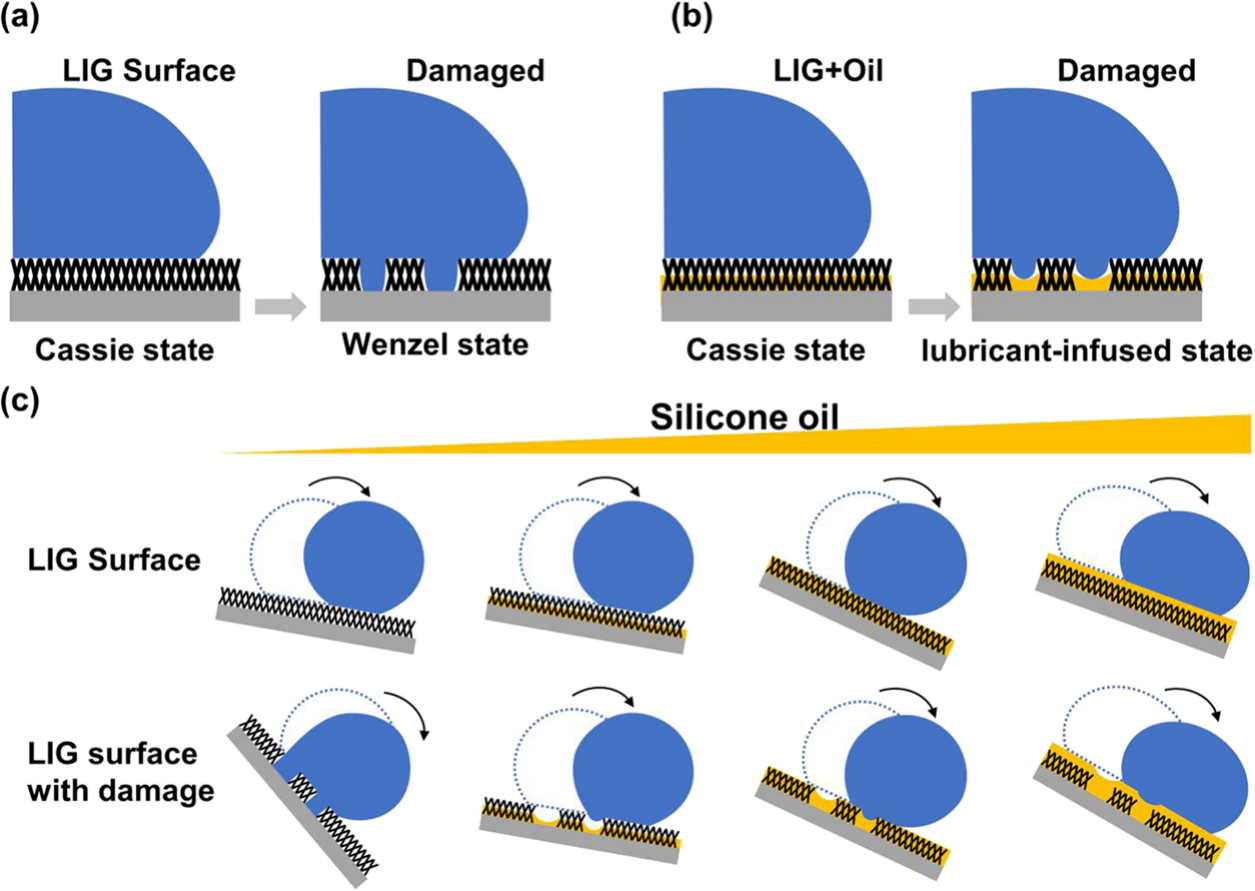
Proposed working mechanism of the dual-functional superhydrophobic-lubricant-infused surface. (a) Schematic illustration of wetting state transitions: conventional superhydrophobic surfaces (without lubricant) undergo irreversible Cassie to Wenzel transition upon damage. (b) Schematic illustration of wetting state transitions: Optimally lubricant-infused surfaces transition from the Cassie–Baxter state to a functional lubricated state upon damage, maintaining liquid repellency. (c) Comparative analysis of droplet behavior on LIG surfaces with varying silicone oil loading (left to right: no oil, little oil, almost full oil, and full oil coverage) in both intact (upper row) and damaged (lower row) conditions, demonstrating how optimal oil loading enables effective self-healing and maintained mobility after surface damage.

Figure 6c further elaborates on the role of oil loading. Insufficient oil (left panels) offers little protection, and the damaged surface behaves like a conventional SH surface, leading to pinning. Excessive oil (right panels) may diminish the initial SH characteristics (potentially higher initial SA) and might not significantly improve healing compared to the optimal loading. The optimal oil loading (e.g., 2.9 mg/cm^2^, middle panels) strikes a balance: it preserves excellent initial SH properties while providing a sufficient, readily available lubricant reservoir for effective self-healing upon damage. This synergistic interplay among the robust, porous LIG scaffold, the trapped air pockets, and the mobile lubricant reservoir allows the surface to tolerate significant physical damage while maintaining high liquid repellency, significantly outperforming surfaces relying solely on either SH or LIS principles, especially regarding durability and self-healing against localized defects.

## Conclusions

In conclusion, we have developed and characterized a novel hybrid liquid-repellent surface by strategically integrating laser-induced graphene (LIG) hierarchical structures with a controlled silicone oil infusion. This work demonstrates a synergistic approach that effectively harnesses the benefits of both superhydrophobic (SH) and lubricant-infused surface (LIS) technologies while mitigating their respective limitations, particularly the mechanical fragility of SH surfaces.

Our systematic investigation revealed a critical dependence of the wetting behavior and robustness on the infused lubricant loading. An optimal silicone oil loading was identified, which maintains excellent static water repellency and good dynamic repellency characteristics of SH surfaces while simultaneously imparting significant damage tolerance and self-healing capabilities. We elucidated a lubricant-mediated self-healing mechanism: upon physical damage to the LIG structure, the infused oil rapidly wicks to the compromised area, transitioning the local wetting state from a vulnerable Cassie–Baxter state to a robust, slippery lubricant-infused state. This localized transition effectively prevents water droplet pinning and preserves overall liquid repellency, even across damaged regions up to 2 mm in width, a feat unachievable with conventional LIG-based SH surfaces. The enhanced durability was further corroborated by droplet impact tests on model lubricant-infused soot surfaces.

The significance of this work lies in the demonstration of a practical strategy to overcome the durability challenge inherent in many SH surfaces through intelligent lubricant integration. The resulting hybrid surface architecture offers a unique combination of high repellency, robustness against physical damage, and intrinsic self-healing. These attributes make our LIG-lubricant hybrid surfaces highly promising candidates for a wide range of demanding applications, where sustained liquid repellency in harsh environments is critical. Potential applications include durable self-cleaning coatings for various substrates, advanced anti-icing surfaces, efficient oil–water separation technologies, and reliable microfluidic devices requiring long-term operational stability. Future research could focus on further optimizing the lubricant-structure interaction, exploring long-term lubricant stability under various operational stresses, and scaling up the fabrication process for broader technological implementation.

## Supplementary Material

















## Notes

Water droplet behavior on 1 mm strip damaged surfaces; left: Silicone oil-infused surface (2.9 mg/cm^2^) shows water droplet rolling off smoothly across damaged region; right: Noninfused surface shows droplet adhesion and pinning at damaged area; this confirms the self-healing effect of silicone oil infusion shown in Figure 3d (Video S1) (AVI)
Candle soot durability test (Figure 4); left: Oil-infused surface maintains water repellency despite 30+ droplet impacts; right: Noninfused surface shows rapid degradation with soot layer detachment after ∼20 impacts and complete failure of water repellency (Video S2) (AVI)
High-speed water jet impact on pristine LIG superhydrophobic surface; the video demonstrates the partial loss of the underlying air layer upon 1.6 m/s water jet impact, evidenced by the disappearance of silvery reflective appearance and exposure of black LIG substrate; large water droplets remain on the surface after impact, with some regions showing complete loss of air layer beneath (Video S3) (AVI)
High-speed water jet impact on the LIG surface with silicone oil; the video shows no observable loss of air layer during impact and no large droplet residue remaining after the test, demonstrating the protective effect of the hybrid surface design (Video S4) (AVI)
Long-term durability testing of PDMS-oil lubricant-infused surface under continuous water droplet impact; the video shows progressive degradation with decreased sliding velocity and eventual droplet pinning after approximately 30 impacts (Video S5) (AVI)
Long-term durability testing of TiO_2_-based lubricant-infused surface under continuous water droplet impact; the video shows progressive degradation with decreased sliding velocity and eventual droplet pinning after approximately 60 impacts (Video S6) (AVI)
Long-term durability testing of LIG-oil hybrid surface under continuous water droplet impact; the video demonstrates excellent droplet mobility maintenance without any pinning behavior throughout extended testing (Video S7) (AVI)
Extended durability comparison showing LIG-oil hybrid surface performance after 2500 water droplet impacts, maintaining consistent droplet sliding behavior without degradation (Video S8) (AVI)
